# Evolution of temporomandibular joint reconstruction: from autologous tissue transplantation to alloplastic joint replacement

**DOI:** 10.1038/s41368-024-00339-3

**Published:** 2025-03-10

**Authors:** Hanghang Liu, Liwei Huang, Shibo Liu, Linyi Liu, Bolun Li, Zizhuo Zheng, Yao Liu, Xian Liu, En Luo

**Affiliations:** 1 State Key Laboratory of Oral Diseases & National Center for Stomatology & National Clinical Research Center for Oral Diseases, West China Hospital of Stomatology, Sichuan University, Chengdu, China; 2 Maine Medical Center Research Institute, Maine Medical Center, Scarborough, ME USA

**Keywords:** Oral diseases, Reconstruction

## Abstract

The reconstruction of the temporomandibular joint presents a multifaceted clinical challenge in the realm of head and neck surgery, underscored by its relatively infrequent occurrence and the lack of comprehensive clinical guidelines. This review aims to elucidate the available approaches for TMJ reconstruction, with a particular emphasis on recent groundbreaking advancements. The current spectrum of TMJ reconstruction integrates diverse surgical techniques, such as costochondral grafting, coronoid process grafting, revascularized fibula transfer, transport distraction osteogenesis, and alloplastic TMJ replacement. Despite the available options, a singular, universally accepted ‘gold standard’ for reconstructive techniques or materials remains elusive in this field. Our review comprehensively summarizes the current available methods of TMJ reconstruction, focusing on both autologous and alloplastic prostheses. It delves into the differences of each surgical technique and outlines the implications of recent technological advances, such as 3D printing, which hold the promise of enhancing surgical precision and patient outcomes. This evolutionary progress aims not only to improve the immediate results of reconstruction but also to ensure the long-term health and functionality of the TMJ, thereby improving the quality of life for patients with end-stage TMJ disorders.

## Introduction

The temporomandibular joint (TMJ) functions as a complex sliding-hinge mechanism, facilitating the articulation between the mandible and the temporal bone of the skull. Diagnosing acute or chronic extra-articular temporomandibular disorders (TMD) relies on identifying dysfunctions or discomfort in the masticatory muscles and the jaw, specifically within the TMJ region. Notably, the etiology of most TMD cases is attributed to muscular factors, and 85%–90% of these patients can be treated effectively with non-invasive interventions.^1^ However, in situations where end-stage TMD occurs within the joint, more invasive interventions become necessary to restore the functional integrity of the mandible.^2^

TMD are characterized by the emergence of functional and pathological disturbances accompanied by discomfort around the TMJ. Commonly, these disorders encompass auditory manifestations within the TMJ, such as clicking, alongside restricted mandibular mobility, pain in the ear and neck regions, and headaches.^3^ Clinically, 95% of individuals exhibit manifestations correlating with extra-articular TMD. Within this cohort, ~50% display complications unrelated to the TMJ itself. Consequently, this delineates that ~45% of cases represent genuine extra-articular, muscle-related TMD. These particular instances typically receive management through non-surgical avenues, including pharmacotherapy, the application of oral appliances, or physiotherapeutic interventions, thereby obviating the need for invasive treatment methodologies. A mere 5% of individuals diagnosed with TMD present with intra-articular variations. These cases are typically associated with a range of complex pathologies, including developmental anomalies, neoplastic conditions, traumatic arthritis, and end-stage ankylosis, frequently necessitating the implementation of invasive therapeutic interventions.^4^ Among the available intra-articular TMD management options, arthroscopy represents a minimally invasive approach that can facilitate the liberation or repositioning of the articular disc,^5^ or execute a discectomy in cases where the articular disc is identified as torn, dislocated, or misshapen.^5^

**Fig. 1:**
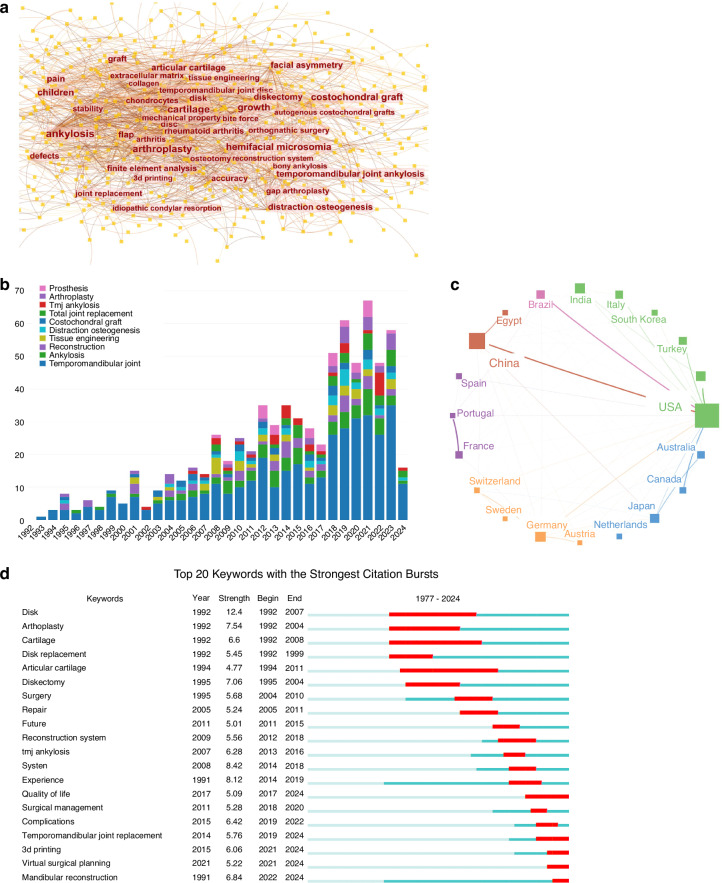
Bibliometric analysis of relevant publications on autogenous or alloplastic TMJ replacements. Using CiteSpace, VOSviewer, and Scimago Graphica for visualization, this figure encapsulates various dimensions of the research landscape. **a** The keyword co-occurrence map illustrates the temporal overlap of key terms appearing at least 15 times, with font size denoting keyword frequency. **b** A bar chart displays the annual distribution of relevant publications from 1992 to 2024, highlighting the top 10 keywords in the field. **c** An international collaboration map identifies countries/regions contributing at least 20 publications. **d** The top 20 keywords featuring strong citation bursts are presented, with a red bar signifying peak citation years

However, in instances where intra-articular disease advances to an end-stage condition, the necessity for joint replacement may arise. End-stage TMJ pathology leads to significant deterioration in both the physiological functionality and structural integrity of the mandible, necessitating total joint replacement (TMJR). This procedure typically involves either autogenous or alloplastic joint replacements. In this study, we conducted a comprehensive bibliometric analysis of publications on the topic of autogenous or alloplastic joint replacements, utilizing the Web of Science Core Collection (WoSCC) database. Our analysis generated an overlay visualization map of keyword co-occurrence, revealing emerging research hotspots such as “growth,” “accuracy,” “3D printing,” and “ankylosis” (Fig. 1a). In addition, we observed a significant increase in the volume of publications in this field over the past decades, from 1977 to 2024 (Fig. 1b). This growth underscores the active engagement of scholars from diverse institutions and countries in TMJR research, highlighting its global impact and the urgent need for advancements in this area (Fig. 1c). Further analysis of keywords with citation bursts and co-cited references over the past five years emphasizes the rising popularity and relevance of “3D printing” and “virtual surgical planning” in recent advancements (Fig. 1d).

**Fig. 2:**
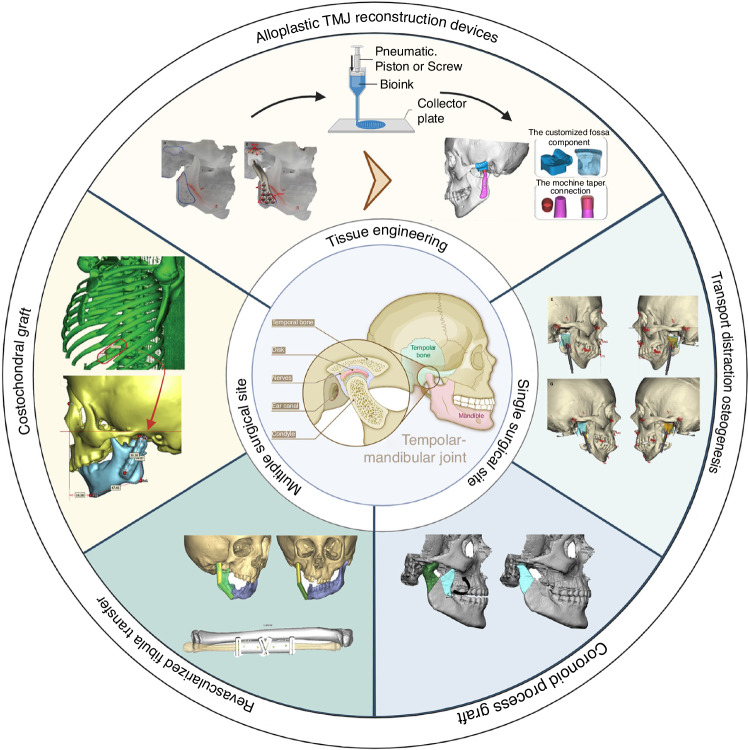
Current application of autologous tissue transplantation and alloplastic joint replacement in TMJ reconstruction. Current application of autologous tissue transplantation and alloplastic joint replacement in TMJ reconstruction. Adapted with permission from refs. ^29,53,111,122,291,297,298^. Copyright © 2023 by the authors; Copyright © The Author(s) 2019; Copyright © 2018 American Association of Oral and Maxillofacial Surgeons; Copyright © 2019 American Association of Oral and Maxillofacial Surgeons; Copyright © 2017 Elsevier Inc; Copyright © 2022 Tanta Dental Journal; Copyright © 2017 Elsevier Inc

Historical records from the early 20th century indicate various sources for autogenous TMJR, such as the costochondral rib, fibula, transport distraction osteogenesis, coronoid process, iliac crest, and sternoclavicular constructs. Among these, the costochondral rib, coronoid process, distraction osteogenesis, and revascularized fibula transfers have become the most common methods.^6^ In the 1960s, Sir John Charnley pioneered the introduction of alloplastic orthopedic joint replacement using metal prostheses.^7^ Over the subsequent six decades, a diverse range of designs and materials have been developed for TMJR, varying from stock TMJ prostheses to more complex patient-fitted and 3D-printed systems. Initially, materials such as stainless steel and cobalt-chromium-molybdenum (Co–Cr–Mo) were commonly applied in TMJR. However, in recent years, there has been a significant shift towards the use of titanium, polyethylene, ceramics, and 3D printing biomaterials due to their growing popularity and potential advantages^6^ (Fig. 2).

This review will concentrate on providing a comprehensive summary of the most frequently utilized techniques in TMJR. In addition, we will discuss the contemporary state-of-the-art pertaining to various TMJR systems. Furthermore, the review will explore the potential of emerging materials that might overcome the existing limitations in the field.

## Autologous tissue transplantation in TMJ reconstruction

In 1908, Bardenheuer pioneered the use of a patient’s fourth metatarsal for mandibular condyle replacement, marking the initial application of autogenous reconstruction in this field.^8^ This approach subsequently established itself as the gold standard for addressing developmental deformities, end-stage TMJ pathology, and ankylosis (Table 1).

**Table 1:** Advantages and limitations for autologous tissue transplantation of TMJR

Autologous Tissue Transplantation	Advantages	Limitations	Recurrence/%
Costochondral grafts	1. Most widely used; 2. With a cartilage cap, mimicking both bone and cartilaginous components; 3. Has intrinsic growth potential; 4. Gross anatomic similarity to the mandibular condyle; 5. Easy accessibility and adaption.	1. Need for second surgical site; 2. Long surgical duration; 3. Poor bone quality, high relapse potential when combined with orthognathic surgery; 4. Unpredictable growth; 5. Possible separation of cartilage from bone.	2–39^19,24,31,49–51,301–304^
Coronoid process graft	1. Avoidance of secondary surgical site and associated donor complications; 2. Enhance mouth opening.	1. No long-term studies; 2. Ankylosed segment may involve the coronoid; 3. No growth center; 4. High bone resorption; 5. Pointed architecture.	2.98–26.7^41,42,49–51^
Revascularized fibula transfer	1. High survival rate; 2. Suitable for large mandibular defects; 3. Tubular shape and densely cortical.	1. Lacks articular cartilage; 2. Donor-site complications including ankle stiffness, instability, and weakness.	2.9–63^66,72,78,305^
Transport distraction osteogenesis	1. No need for interpositional material; 2. Simultaneous correction of soft tissue defect; 3. Shorter surgical duration; 4. Increase vertical height; 5. Avoidance of secondary surgical site and associated donor complications.	1. No potential growth in growing patients; 2. High requirement for patient cooperation; 3. Lengthy procedure.	10–28.6^50,118,119,306^

### Costochondral grafts

**Fig. 3:**
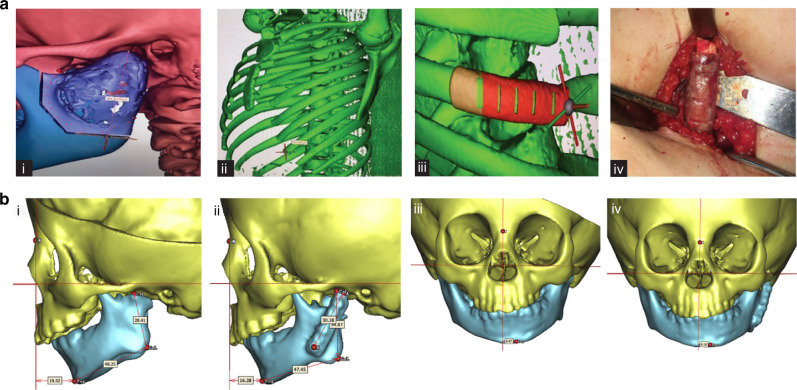
Costochondral grafts used in TMJ reconstruction. **a** Computer-assisted surgical simulation technology and three-dimensional reconstruction of TMJ, the sixth rib, and surgical template to guide accurate costochondral graft cutting.^298^ Copyright © 2022 Tanta Dental Journal. **b** Three-dimensional reconstruction of ramus and costochondral graft with left TMJ ankylosis, pre- and post-surgery.^29^ Copyright © 2017 Elsevier Inc

First described by Gillies in 1920,^9^ ostochondral grafts (CCG) have since become the autogenous bone graft of choice for reconstructing the ramus-condyle unit (RCU), owing to their biological compatibility, limited donor site morbidity, and growth potential.^10,11^ CCGs are believed to possess primary and secondary growth centers, situated at the juncture of the cartilaginous section and bony parts of the graft, mirroring the growth rate of the mandibular condyle.^12^ Among the fifth, sixth, and seventh ribs typically utilized for reconstruction, the sixth rib is the most commonly selected^13^ (Fig. 3).

Initially, CCG was predominantly used in pediatric patients for its potential to accommodate growth in skeletally immature individuals.^14,15^ Despite this advantage, well-documented complications such as resorption, fracture, ankylosis, and unpredictable growth patterns frequently emerge post-grafting.^16–18^ Medra observed a re-ankylosis rate of 9%, graft resorption in 25%, and overgrowth in 4% among patients undergoing CCG for TMJR.^19^ Although autogenous CCG is theorized to grow in tandem with the patient, this growth has often been reported as unpredictable or resulting in ankylosis.^17,18,20–22^ Long-term studies on CCG for TMJ reconstruction reveal excessive growth on the treated side in 54% of patients, with only 38% achieving symmetrical RCU (ramus-condyle unit) four years post-TMJR.^11^ Similarly, another study noted significant short-term improvements in mandibular and facial symmetry in hemifacial microsomia patients; however, a 93% rate of secondary surgery requirement emerged for symmetry maintenance, attributed to prevalent undergrowth a decade post-TMJR with CCG.^23^ In a comprehensive retrospective review of 76 patients who underwent CCG for TMJR, Kent^16^ observed a notably higher complication rate in patients with a preoperative diagnosis of ankylosis, often necessitating additional surgeries. These complications are potentially attributable to inadequate revascularization and micromotion.

A recent systematic review^13^ assessing complications in adolescent patients undergoing CCG for TMJR included 8 studies and a total of 95 included cases. Reported postoperative complications encompassed re-ankylosis (6.32%), insufficient graft growth (22.11%), unpredictable graft overgrowth (13.70%), absence of graft growth (3.20%) and subsequent facial asymmetry (20%). In addition, a related meta-analysis highlighted graft overgrowth in 30.89% of cases, while optimal growth was observed in 55.89% of subjects.^24^ Consequently, employing CCG in young patients for temporomandibular ankylosis reconstruction is associated with a considerable risk of growth abnormalities. The necessity of using cartilage-containing grafts for mandibular growth maintenance and restoration has been recently questioned. To address these challenges, technical modifications have been suggested, including limiting the cartilaginous cap’s thickness to deter overgrowth and lining the glenoid fossa with soft tissue, such as vascularized temporalis fascia,^25–30^ or alternative interpositional materials,^31^ particularly when the native disc is unrecoverable, thereby diminishing the likelihood of re-ankylosis and growth abnormalities.^10,11,32^ Kaban et al.^10^ noted that maintaining 3 to 4 mm of cartilage is sufficient to prevent both ankylosis and overgrowth. Some studies have also advocated for ipsilateral and/or contralateral coronoidectomy to enhance mouth opening.^22,25,27,28^ Despite this, a recent biomechanical analysis revealed that bilateral TMJ reconstruction combined with coronoidectomy for substantial mandibular advancement (≥10 mm) can significantly increase shear force, potentially leading to fractures at the costal-cartilage junction. This suggests that the necessity of coronoidectomy should be thoroughly evaluated before proceeding.^33^ Moreover, advancements in endoscopy have facilitated the use of intra-oral, subangular, and modified preauricular incisions, offering alternatives to the traditional submandibular approach for CCG^34,35^

### Coronoid process graft

The Coronoid Process Graft (CPG) emerged as a prominent grafting option for TMJR, first introduced by Youmans in 1969.^36^ Since its inception, CPG has gained widespread acceptance for addressing TMJ ankylosis and severe mandibular retrognathia, with particular prominence in China.^37–45^ In several of these interventions, interpositional materials such as temporal muscle myofascial flaps,^37,39,46,47^ prosthodontic membrane,^43^ or native articular disc^41^ have been utilized to enhance the outcomes of the grafting procedure.

**Fig. 4:**
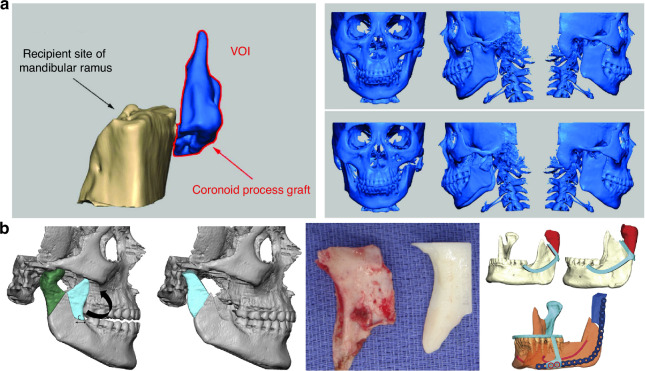
Coronoid process graft used in TMR reconstruction. **a** Preoperative maxillofacial hard tissue structures and surgical simulation of the autogenous coronoid process graft reconstruction for the treatment of unilateral temporomandibular joint ankylosis.^45^ Copyright © 2017 Liu et al. **b** Planned surgical removal of specimen (condyle and ramus) and harvesting and rotation of coronoid-ramus graft, the inverted coronoid graft is secured to the reconstruction plate and fixated distally initially using guide holes.^53^ Copyright © 2019 American Association of Oral and Maxillofacial Surgeons

The nature cortical density of the coronoid process renders it more capable of enduring substantial forces compared to CCG, a feature mirrored in its lower ankylosis rates (2.98%) as opposed to ~8% observed with other graft types.^24,48,49^ Notably, a comprehensive long-term retrospective cohort study revealed a higher likelihood of TMJ ankylosis recurrence in adults treated with CPG (26.7%), compared to those undergoing reconstruction with either CCG or distraction osteogenesis, which reported no recurrence at a 10-year follow-up.^50^ This observed variance may be attributed to factors such as the classification of TMJ ankylosis, the extent of surgical removal of bony fusion, and inadequately lengthy follow-up periods. The study also proposed that resorption of the coronoid process could stimulate osteoblast differentiation and new bone formation within the TMJ biomechanical environment, potentially leading to reankylosis.^50^ Despite these considerations, reconstructions using the coronoid process have been associated with improved masticatory efficiency, bite force, and range of motion compared to other grafting methods.^51^ In addition, incorporating a simultaneous coronoidectomy has been shown to enhance mouth opening,^37^ and employing CPG for TMJR obviates the need for a secondary surgical donor site (Fig. 4a).

The most prevalent complications associated with coronoid grafts are graft resorption (36.3%) and temporary nerve paresis (8.69%)^24,48,49,52^ with the frontal branch of the facial nerve being the most commonly affected. Nonetheless, complete recovery was observed within 3–6 months.^48^ Zhu^41^ reported no occlusal changes due to bony resorption at a 2-year follow-up, whereas Huang’s study^52^ highlighted a significant increase in malocclusion and a more pronounced decrease in ramus height in the CPG group in comparison with the CCG group. A recent meta-analysis revealed that both CCG and CPG grafts for TMJR performed similarly in terms of re-ankylosis rates and postoperative MIO. However, the CPG group exhibited a notably lower relapse rate of 2.98%, while the pooled relapse rate for CCG was ~8%.^24^ A long-term follow-up study demonstrated improved joint function in both pedicled coronoid process grafts on the temporal muscle and autogenous free coronoid process grafts. The latter, however, showed more notable bony resorption and a higher decrease in mandibular ramus height,^42^ suggesting that interpositional tissue may mitigate bony resorption and enhance long-term outcomes in CPG application for TMJR. In addition, Heffez introduced a novel technique for condylar reconstruction involving the rotation of the ipsilateral coronoid process-mandibular ramus by 180° along its horizontal axis to serve as a replacement for the excised condyle, supported by visual surgical planning^53^ (Fig. 4b). However, this method, which demonstrated resistance to resorption and maintained the morphology of the ramus and condyle a limited number of cases, was not recommended for growing patients. Furthermore, visual surgical planning has been proven to be an effective approach for improving the safety and efficacy of condyle reconstruction, particularly in patients with bilateral TMJ ankylosis using CPG, resulting in fewer postoperative malocclusions and facial nerve injuries.^54^

Similar to CCG, the application of CPG in condylar reconstruction prompts a pertinent question, particularly regarding their growth potential and suitability for TMJR in children with unilateral TMJ ankylosis. A study with a 5-year follow-up period explored this potential in adolescent patients who underwent condyle reconstruction using an ipsilateral coronoid process, supplemented by interposed pedicled temporalis fascial flap.^55^ The findings revealed that, post-TMJR with CPG, there was continued growth in both the ramus height and mandibular length (25% increase in height and 26% increase in length), albeit the growth deficit was not fully compensated. Specifically, the increase in the ramus height on the affected side was 47% less, and the mandibular length on the affected side was 27% shorter in comparison to the healthy side.^55,56^ Consequently, a second surgical intervention may be necessary for adolescent patients undergoing TMJR with CPG.

### Revascularized fibula transfer

Since its inaugural application in mandibular reconstruction in 1989,^57^ the revascularized fibula transfer (RFT) has emerged as the cornerstone for repairing critical-sized segmental defects of the mandible—predominantly following oncologic resection, trauma, or osteonecrosis—over the past several decades.^6,58–70^ The fibular free flap technique offers considerable versatility, enabling the reconstruction of any mandibular segment through precisely angled osteotomies. The majority of patients have reported excellent bony contours, the ability to resume oral feeding, achieve esthetically pleasing results, and maintain clear speech. Nevertheless, the accompanying soft tissue deficit, particularly noticeable in the buccal and parotid areas due to fat loss, often leads to facial asymmetry.^59^

Reconstructing mandibular defects that involve the condyle present a significant challenge, particularly in restoring the function of the TMJ using RFT. Achieving precise alignment of the bone graft is critical for the full restoration of joint function. In this context, VSP and computer-aided design and manufacturing (CAD/CAM) technology emerge as valuable tools for the accurate reconstruction of mandibular condyle defects using RFT, potentially eliminating the need for additional procedures. Although initial studies have not demonstrated functional superiority of CAD/CAM-assisted TMJ reconstructions using RFT over traditional techniques, these advanced methods may facilitate more precise reconstructions of the TMJ.^71^ Moreover, they offer the potential to significantly reduce preoperative irradiation volume and decrease the number of required intraoperative osteotomies.^69–74^

**Fig. 5:**
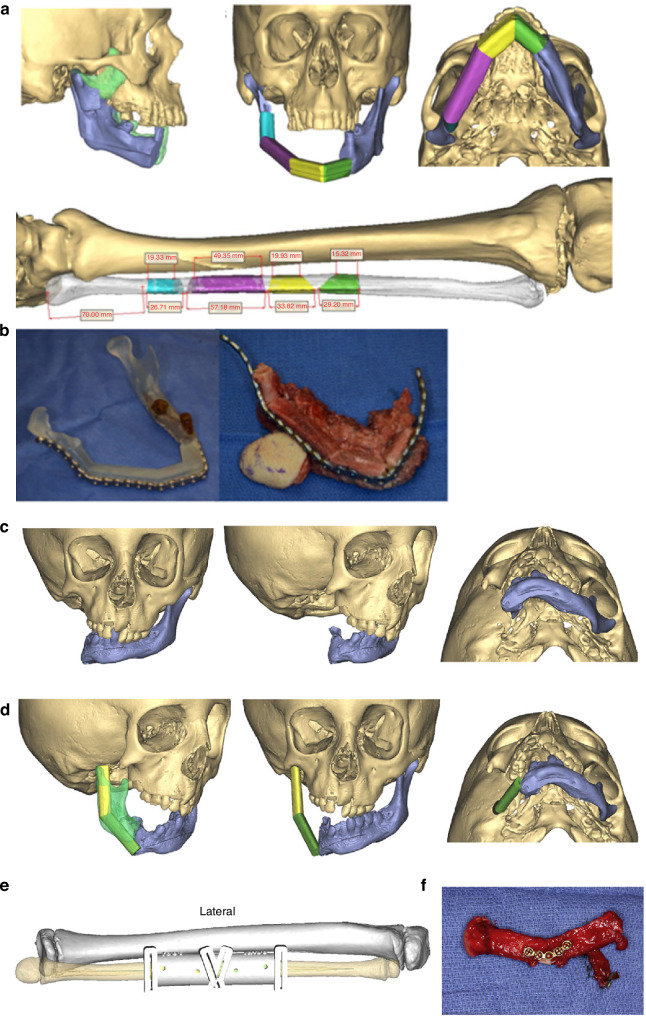
Revascularized fibula transfer used in TMJ reconstruction. **a** Planned reconstruction with 4 fibular segments and grafting the condylar head onto the fibula. **b** Pre-bent reconstruction bar contoured to preoperative model (left) then attached to the fibular free flap following osteotomies (right).^299^ Copyright © The Author(s) 2016. **c**, **d** A free-fibula flap is virtually positioned with one osteotomy to facilitate the planned mandibular position and mimic the contralateral mandibular contour, which has been directly transposed onto the glenoid fossa. **e** A surgical guide is fabricated to dictate the length and internal osteotomy of the fibula flap. **f** The fibula flap is harvested and an AlloDerm (Allergan, Inc, Parsippany, NJ) cap is applied to serve as the articulating surface.^122^ Copyright © 2017 Elsevier Inc

Condylar reconstruction via RFT transfer typically employs three approaches: grafting the condylar head onto the fibula when oncologically viable^60^ (Fig. 5a, b), or direct placement of the transfer into the glenoid fossa, with or without prior contouring^59,61^ (Fig. 5c–f). The preservation of the condylar head, recognized as a critical growth center for the mandible in pediatric patients, is crucial to circumventing long-term sequelae such as malocclusion and associated maxillary deformity.^60^ Nonetheless, recent research has highlighted an increased risk of locoregional recurrence when preserving the condyle in cases of posterior mandibular lesions.^75^ Remarkably, instances of ankylosis have not been reported even when the fibula’s distal end is directly inserted into the glenoid fossa without contouring, under an intact articular disc to serve as a neocondyle, across 1–2 years of follow-up.^59,61,63,68,76,77^ Furthermore, evidence of new condyle regeneration characterized by cartilaginous tissue formation has been documented in RFTs lacking initial cartilage, at one-year post-implantation.^64^

The potential for severe complications, such as ankylosis and functional limitations of the TMJ, warrants careful consideration when employing RFT for condylar reconstruction. Future research should meticulously assess the impact of the TMJ disc’s presence on surgical outcomes. The preservation and reattachment of the TMJ disc and the lateral pterygoid muscle within the glenoid fossa may sustain TMJ’s normal functionality and diminish the likelihood of re-ankylosis.^65,77^ Notably, Resnick observed a 63% re-ankylosis rate over a 4-year follow-up in patients undergoing RFT for ramus and condyle construction aimed at treating hemifacial microsomia, especially Kaban-Pruzansky type III mandibular deformities.^78^ A significant factor influencing ankylosis incidence was identified as the application of a sagittal ramus osteotomy on the contralateral side. This procedure facilitates rotational adjustment and mitigates the force exerted by the fibula against the skull base in skeletally mature patients, thereby lowering the ankylosis rate.

RFT transfer presents similar challenges to CCG and CPG for TMJ reconstruction within the growing facial skeleton. Recent studies^64,79^ have highlighted the absence of growth potential in RFT, suggesting that growing patients undergoing TMJR with RFT may require a bilateral sagittal split osteotomy upon reaching skeletal maturity. Furthermore, there appears to be an increased risk of ankylosis post-TMJR using RFT, particularly in skeletally immature patients at the time of surgery.^78^ However, employing RFT that includes the proximal epiphysis—comprising the growth plate and articular surface—and positioning it towards the articular fossa of the temporal bone has demonstrated promising functional and esthetic outcomes in adolescent patients. Such an approach has resulted in the growing reconstructed RCU in harmony with the contralateral side, eliminating the need for surgical revisions one-year post-operation.^80^

### Transport distraction osteogenesis

Distraction osteogenesis (DO) represents a pivotal technique for TMJR, especially in scenarios lacking suitable bone graft options. DO induces bone regeneration through gradual separation of surgically divided bone segments, following a posterior mandibular vertical ramus osteotomy.^81^ Essentially, a vertical growth vector is established between the stable proximal mandible and the osteotomized posterior mandibular segment, guiding the osteotomized segment toward the glenoid fossa to cultivate a neo-condyle.^82^ Originated by Ilizarov in the 1950s for long bone defect reconstruction, this method was adapted for craniofacial applications in the 1990s, showcasing its versatility.^83,84^ DO can be categorized into elongation DO (EDO), which extends existing bone, and transport DO (TDO), which bridges segmental defects. The application of DO in RCU reconstruction offers several advantages: it allows precise control over the direction and magnitude of bone elongation, facilitating concurrent soft and hard tissue expansion; it obviates the need for bone grafting, thereby reducing donor site morbidity; and it enhances structural stability.^85^

**Fig. 6:**
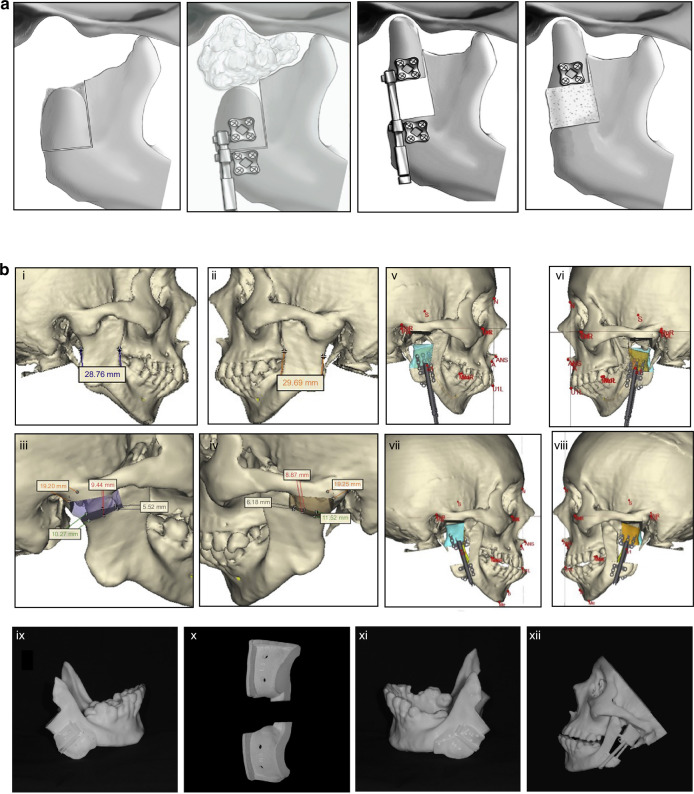
Transport distraction osteogenesis used in TMJ reconstruction. **a** Scheme showing the process of Transport distraction osteogenesis to reconstruct TMJ.^300^ Copyright © 2008 American Association of Oral and Maxillofacial Surgeons. **b** Scheme showing the process of virtual treatment planning, repositioning of bony segments by distraction osteogenesis and series of surgical templates used to transfer the virtual plan to actual surgery.^111^ Copyright © 2018 American Association of Oral and Maxillofacial Surgeons

Stucki-McCormick initially reported the clinical use of extraoral TDO for RCU reconstruction in humans, marking a significant advancement in the field.^85,86^ The procedure involves a reverse-L osteotomy extending from the sigmoid notch to the posterior border of the mandible, performed either to release bony ankylosis or following tumor resection. An external transport distraction device is then affixed, facilitating the superior advancement of the segment by 1 to 2 mm daily until it aligns with the glenoid fossa (Fig. 6a). In cases where the articular disc is absent, a temporalis muscle and fascia flap often serve as interpositional materials to bridge the gap created by arthroplasty.^87^ Post-distraction, new cortical layer formation on the articular surface and the development of a pseudodisk have been observed, indicating the remodeling capabilities of the bone under distraction forces.^86^ Hikiji et al. further identified cartilaginous cells and subsequent ossification within the cartilaginous matrix on the transport disk’s upper surface in rat models, suggesting that these cells likely originate from undifferentiated mesenchymal cells in the bone marrow and internal periosteum, triggered by the trauma’s biological signals.^88^ The application of gap arthroplasty and extraoral TDO for TMJ reconstruction has since become prevalent for patients with TMJ ankylosis, including those with micrognathia, across both skeletally mature^81,87,89–98^ and growing populations.^99–108^ This two-staged surgical approach has yielded substantial functional and esthetic improvements over 1–4 years of follow-up. However, the cutaneous scars from the extraoral distraction, often hypertrophic and conspicuous, prompted the exploration of intraoral TDO devices^87,103,109^ and single preauricular incision TDO^94^ as alternatives to minimize scarring. Recent studies also incorporate simultaneous genioplasty,^93,98^ and employ VSP and CAD/CAM surgical assistant system^92,110,111^ to further enhance facial esthetics and respiratory function, showcasing the evolution of TDO techniques in TMJR (Fig. 6b).

A comparative study examining different TMJR grafts revealed no significant differences in mean mouth opening and excursive movements between the TDO and sternoclavicular graft groups. This finding was echoed in another randomized trial and meta-analysis, which demonstrated that RCU reconstruction using either CCG or TDO effectively forms a neocondyle, maintains occlusion, and corrects facial asymmetry.^112,113^ However, the TDO group exhibited significantly greater mean condylar resorption over the follow-up period.^114^ A more recent meta-analysis showed significant postoperative improvements in mouth opening in both the TDO and CCG groups, with the analysis favoring TDO for joint reconstruction. The incidence of postoperative re-ankylosis was up to 6.1% lower in the TDO group compared to the CCG group.^115^

Notably, substantial relapse rates in the length of the corpus (25%) and the height of the RCU (26%–87%) post-distraction were reported by several researchers.^97,116,117^ A recent long-term follow-up study highlighted that while TDO offers stable short-term esthetic improvements within the first postoperative year, significant reductions in the reconstructed RCU and a 10% recurrence rate of TMJ ankylosis were observed 7–12 years post-surgery.^118^ This decrease in bone length may be attributed to remodeling processes at the gonion and pogonion, influenced by alterations in soft-tissue muscle pull dynamics on the mandible. To mitigate the risk of re-ankylosis post-gap arthroplasty and TDO, modifications in distraction devices have been explored. The introduction of the Matthews craniomandibular fixation device^119^ and dual distraction device^120^ reported successful maintenance of facial symmetry, with no instances of relapse or re-ankylosis during the follow-up period. These advancements underscore the continuous evolution of TMJR techniques, aiming to enhance long-term outcomes and patient satisfaction.

The application of TDO in reconstructing the RCU has been posited to retain the growth potential of the regenerated ramus and condyle, allowing it to develop in harmony with the contralateral, untreated side. Studies have shown that the neo-condyle formed through TDO does not exhibit statistically significant differences when compared to the natural condyle on the non-operated side.^6,99,100,103,105–108,112^ Despite these promising findings, the postoperative growth potential in growing patients remains uncertain, with reports of varying degrees of facial deformity and unpredictable mandibular growth following TMJ arthroplasty.^121,122^ Xiao’s research further underscores this uncertainty, revealing a 16.7% increase in the mandibular asymmetry difference ratio post-TMJR using TDO in adolescent patients, indicating instability in the heights of reconstructed condyles over the long term and a tendency toward asymmetry.^104^ This raises critical questions about the appropriateness of simultaneously performing ankylosis release and mandibular distraction in patients without clear indicators of potential growth. It prompts a reconsideration of whether mandibular distraction osteogenesis should be staged as a secondary procedure following gap or interpositional arthroplasty to address residual asymmetry or retrognathism once skeletal maturity is reached.^123^ Further research is imperative to navigate these considerations and optimize treatment strategies for growing patients.

## Transition to alloplastic TMJR

### Development of alloplastic TMJR devices

Autogenous grafts, while commonly employed, are associated with several disadvantages, including the necessity for an additional surgical site, donor site morbidity, the risk of graft over- or undergrowth, potential for graft fracture or resorption, and extended surgery duration.^124^ In contrast, alloplastic total joint replacement has been recognized as a promising strategy for managing unilateral or bilateral TMJ ankylosis, juvenile idiopathic arthritis, and idiopathic condylar resorption,^125–128^ offering an innovative alternative to conventional techniques. A 1-year follow-up comparative study found no significant difference in maximal interincisal opening between the prosthetic TMJR group and the CCR graft group. Similarly, changes in preoperative and postoperative pain scores were also insignificant between the groups.^129^ However, longer-term evidence indicated that patients treated with alloplastic TMJR experienced greater improvement and fewer complications compared to the CCR group. In addition, more patients in the autogenous group required reoperation.^130,131^ The complications in the alloplastic TMJR group were generally self-limited, including transient facial nerve weakness, temporary malocclusion, or pain during maximum opening. In contrast, the CCR group experienced issues such as re-ankylosis, overgrowth, malocclusion, and minor infections.^131^ A subsequent meta-analysis revealed a significant reduction in pain with alloplastic reconstruction compared to the CCR group.^132^ Another recent meta-analysis also suggested that interpositional gap arthroplasty using autogenous materials and reconstruction with either autogenous grafts or alloplastic prosthetic implants yielded comparable clinical outcomes in the management of TMJ ankylosis.^130,133^ In addition, finite element analyses have indicated that alloplastic TMJ prostheses distribute stress and strain evenly across the alloplastic and contralateral natural joints, minimizing adverse effects on the natural joint.^134^

In 1965, Christensen pioneered the development of the first total TMJ implant, initially combining a Vitallium fossa with a standardized cast Vitallium ramus component featuring a polymethylmethacrylate (PMMA) condylar head, secured with cement.^135^ However, the application of PMMA cement was subsequently discontinued due to PMMA fragmentation under functional loading, which compromised the integrity of the prosthesis. In 1977, Momma introduced another approach, and subsequently, Kent developed a prosthetic design combining a metal condyle with a Teflon-coated glenoid fossa for TMJR.^136^ Nonetheless, this innovation faced setbacks when the FDA in the US retracted its approval and recommended the removal of these implants due to particle shedding and the ensuing foreign body giant cell (FBGC) reaction.^6^ This response exacerbated the deterioration of any autogenous graft materials in proximity to the failed Proplast-Teflon implants.^137^ The setbacks experienced with Kent’s Teflon-based implants paved the way for significant advancements in TMJR. Leveraging insights from long-bone joint replacements, the articulating Teflon layer was substituted with ultra-high molecular weight polyethylene (UHMWPE) in 1986.^138^ This marked a pivotal shift towards the use of titanium and Co–Cr–Mo alloys in combination with UHMWPE. These materials form the basis of most FDA-approved TMJR systems available today for patients with skeletal maturity. However, the success of these implants depends on the availability of adequate host bone for secure fixation and stabilization of the components.

**Fig. 7:**
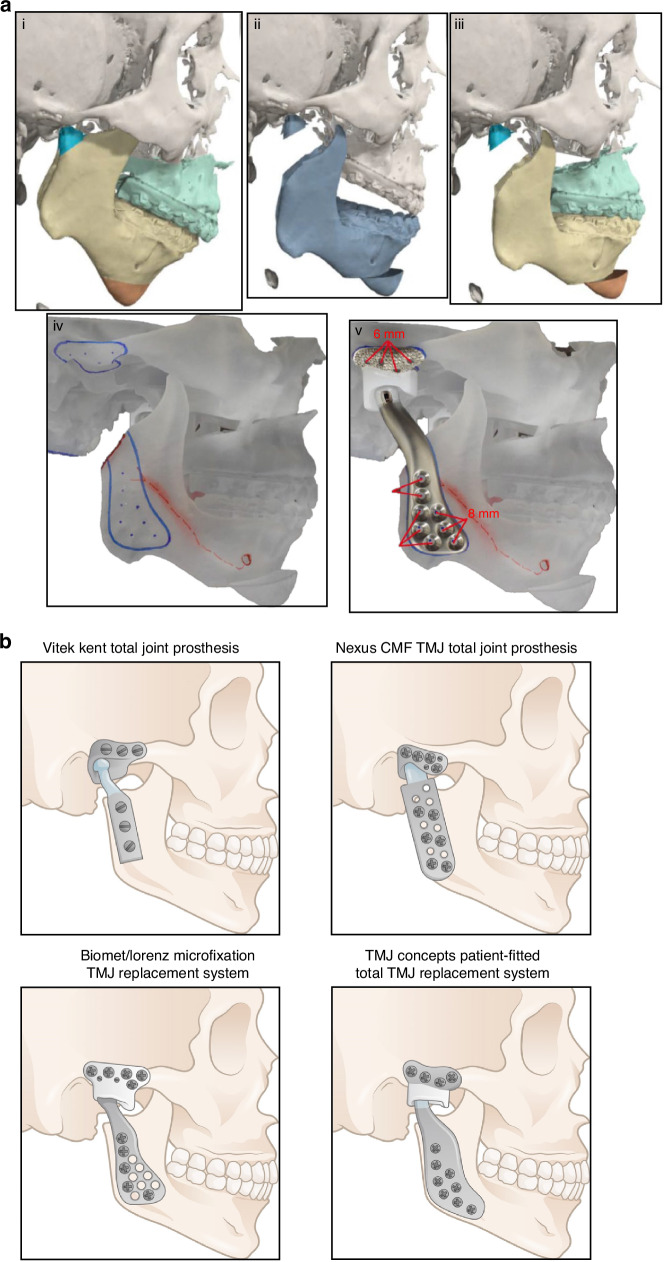
Steps for fabrication of a custom alloplastic TMJ prosthesis and the development of current available commercial alloplastic TMJ prosthesis. **a** Steps for fabrication of a custom alloplastic TMJ prosthesis. Virtual preoperative position→Virtual condylectomy cuts to allow adequate space for the TMJR→Virtual final position producing acceptable facial profile→Printed STL model with initial design of custom alloplastic TMJ prosthesis→Custom alloplastic TMJ prosthesis device with manufacturer recommendations for screw length.^297^ Copyright © 2023 by the authors. **b** The development history of alloplastic TMJR devices and total replacement temporomandibular joints had been approved by FDA

In 1989, LG Mercuri of TMJ Concepts pioneered the development of the first CAD/CAM patient-specific TMJR prosthesis, based on maxillofacial computed tomography scans. This custom approach received FDA investigational device approval in 1996 and was introduced for patient use in 1997.^139^ The TMJ Concepts system features a pure titanium mesh-backed UHMWPE fossa component and a ramus made of cp titanium or wrought Ti–6Al–4V alloy, with a Co–Cr–Mo condyle head and titanium alloy screws (Fig. 7a). This design aimed to mitigate the FBGC reactions associated with Proplast-Teflon implants and address issues of fit, fixation, and long-term stability inherent to stock implants.^140^ Following this, Zimmer Biomet introduced a custom TMJR device employing an all-UHMWPE fossa component, Co–Cr–Mo ramus and condyle components with plasma-sprayed titanium coating, and Ti–6Al–4V alloy screws, which has been FDA approved and demonstrated long-term clinical safety and effectiveness.^141–145^ Despite the inability of alloplastic TMJR to fully replicate natural TMJ function—as indicated by restricted mandibular movement,^146^ long-term studies have consistently showcased its effectiveness. In several retrospective 10-year follow-up studies, Rikhotso et al.,^147^ Rajkumar et al.,^148^ and Leandro et al.^149^ demonstrated that TMJ alloplasts provide satisfactory clinical and functional outcomes for patients with end-stage TMJ diseases. These studies highlighted significant improvements in patients with ankylosis, evidenced by enhanced maximum mouth opening, better chewing ability, improved quality of life, and reduced pain. Likewise, Gerbino et al. reported that TMJ reconstruction using both stock and custom-made devices resulted in improved occlusion and quality of life over a 12-year follow-up period. Their findings underline the long-term effectiveness and reliability of these reconstructive approaches in managing severe TMJ conditions.^150^ These studies have conclusively demonstrated the positive impact of alloplastic TMJR, highlighting significant decreases in chronic pain and substantial improvements in mandibular function, mouth opening, and quality of life post-treatment.^149,151–163^

Bütow et al. and Hoffman initiated the development of a titanium nitride TMJ replacement system, which was introduced in 1994. This innovation involved treating both the fossa and condylar components with nitride to enhance material hardness and improve wear characteristics.^164,165^ Despite these advancements, the Hoffman TMJ replacement system did not secure FDA approval, leading to the cessation of its production. Concurrently, the Nexus CMF and TMJ Concept teams developed a metal-on-metal TMJR system featuring a Co–Cr alloy for the condylar head, ramus, and fossa. Early clinical trials of this system yielded promising results, demonstrating lower wear rates than metal-on-acrylic counterparts and satisfactory clinical outcomes, which facilitated FDA approval in 2001.^166–168^ Nevertheless, long-term follow-up studies revealed serious complications such as metallosis, osteolysis, and implant failure, prompting the FDA to revoke its approval in 2015 and halt production.^169^ This decision underscores the complexities of wear dynamics in TMJR systems, noting that total wear volume in metal-on-metal prostheses can be substantially lower than that observed in metal-on-UHMWPE implants^170^ (Table 2). To address this issue, the TMJ concept incorporates a secure attachment for the fossa’s articulating surface, which consists of UHMWPE bonded to a titanium base mesh. This design may reduce the potential for point contact between metals and subsequent wear. Several long-term investigations have demonstrated that this modified system continues to function effectively, with patients showing significant improvements in TMJ pain, jaw function, the ability to chew solid food, and quality of life.^143,171^ Moreover, the Groningen TMJ prosthesis, initially utilized as a stock device, which was subsequently developed in vitro and later applied clinically. This device features a UHMWPE disc placed between the zirconium condylar and the zirconium surface of the cranial prosthesis. However, an 8-year follow-up study revealed that while there was a significant decline in mandibular function impairment scores compared to baseline, the prosthesis had limited effects on maximum mouth opening, function, and pain.^172^ Consequently, metal-on-UHMWPE TMJR devices have become one of the most popular alloplastic TMJ systems.

**Table 2:** The development history of alloplastic TMJR devices and total replacement temporomandibular joints currently being produced or developed

Surgeon/System	Fossa	Condyle & Ramus	Screws	Design	Year of introduction
Christensen^307^	Co–Cr	Co–Cr with PMMA condyle	N/A	Stock	1965
Kiehn^308^	Co–Cr with PMMA cement	Co–Cr	N/A	Stock	1974
Morgan^309^	Co–Cr	Co–Cr with acrylic condyle	N/A	Stock	1976
Momma^310^	Co–Cr	Co–Cr	N/A	Stock	1977
Kummoona^311^	Co–Cr	Co–Cr with PMMA cement	N/A	Stock	1977
Vitek–Kent^138,312^	Teflon and Proplast	Co–Cr	N/A	Stock	1983
	Proplast and UHMWPE	Co–Cr	N/A	Stock	1986
Techmedica^313^	Titanium and UHMWPE	Titanium with Co–Cr–Mo condyle	N/A	Custom	1989
Butow^314^	Ti nitride alloy	Ti nitride alloy	N/A	Stock	1992
Hoffman and Pappas^164^	Titanium and UHMWPE	Titanium with nitride coated condyle	Ti–6Al–4V	Custom	1993
Biomet System^141,144,145,150,154,161,173–175,179,182,185,186,192,203,204,248,249,315–317^	UHMWPE	Co–Cr–Mo	Ti–6Al–4V	Stock/Custom	2000
Nexus CMF System^166,167,318,319^	Co–Cr–Mo	Co–Cr–Mo	Co–Cr–Mo	Stock	1995
TMJ Concepts System^128,143,157,160,161,168,185,187,195,203,205,238,320–322^	Cp Ti (with UHMWPE articulating surface)	Co–Cr–Mo	Ti–6Al–4V	Custom	1999
Groningen system^172,323^	Titanium and zirconia	Titanium with zirconia condyle	Interpositional disc: UHMWPE	Custom	1999
	Ti alloy-backed UHMWPE	Zr condyle 3D printed Ti alloy ramus (DMLS)	N/A	Custom	2012
Belgium-system^324^	Ti alloy - UHMWPE	3D printed Ti alloy	N/A	Custom	2007
Germany-system^325,326^	All UHMWPE	Co–Cr	N/A	Stock	2008
India-system^327,328^	All UHMWPE	3D printed all Ti alloy	N/A	Custom	2014
	Stainless-steel-backed UHMWPE	Stainless steel	N/A	Stock	2009
	Stainless steel	Stainless steel	N/A	Custom	2009
Brazil-system^329–331^	All UHMWPE/Metal-backed UHMWPE	3D printed Co–Cr–Mo condyle Ti alloy ramus (DMLS)	N/A	Custom	2014
France-system^332^	Stainless steel and zirconium	Stainless steel and zirconium	N/A	Custom	2016
Poland-system^289^	All UHMWPE	3D printed Ti alloy (DMLS); Ti alloy	Ti–6Al–4V	Custom	2017
Australia-system^285,287^	All UHMWPE	3D printed Ti alloy	Ti–6Al–4V	Custom	2017
China-system^333^	All UHMWPE	3D printed Ti alloy	Ti–6Al–4V	Custom	2017
Iraq-system^334^	Zr-Nb alloy	Zr-Nb alloy	Ti alloy	Stock	2018
Italy-system^335^	UHMWPE layer coupled with Ti alloy	3D printed Ti alloy	Ti alloy	Custom	2021

*DMLS* direct metal laser sintering

### Limitations for alloplastic TMJR

#### Stock TMJR devices vs. customized TMJR devices

Stock TMJR devices, while immediately accessible, present limitations regarding size and shape variability.^173^ These constraints necessitate adapting the patient’s anatomy to the prosthesis, particularly in individuals with a short ramus, posing potential challenges.^174,175^ To date, stock devices have demonstrated a survival rate of 96% at three years, 94% at five years, and 86% at ten years.^142,176^ In contrast, custom-designed TMJR devices, which constitute over 75% of the global production, have shown to offer benefits in terms of surgical efficiency and long-term quality of life improvements, based on subjective and objective outcomes over 20+ years.^143,177,178^ Custom TMJR devices are recommended as the standard of care in cases of significant anatomical deviations or substantial changes in mandibular position, such as those necessitating concurrent orthognathic surgery, or in patients with multiple prior surgeries.^142,171,179–181^ VSP has emerged as a reliable method for preoperative surgical planning and execution, enhancing accuracy and precision when utilizing custom TMJ prostheses.^150,182–184^ This approach aims to optimize surgical outcomes. Nevertheless, recent systematic reviews and clinical trials have revealed that both stock and custom TMJR devices significantly improve diet consistency and mouth opening, with no notable differences in outcomes between the two types.^150,185–190^

Onoriobe et al. highlighted a 38% increase in alloplastic TMJR cases from 2005 to 2014.^191^ As of 2023, 19 countries have produced 37 TMJR devices, including 6 stock and 31 custom models, with 10 of these devices being produced through additive manufacturing. Among the three FDA-approved alloplastic TMJR systems (Fig. 7b), TMJ Concepts, Zimmer Biomet, and Nexus CMF—no comprehensive, well-designed controlled prospective studies have distinguished one system as superior. Only one study has suggested that Chinese standard TMJ prostheses offer comparable efficacy and stability to the Zimmer Biomet TMJR system.^192^ And in 2017, in a meta-analysis involving 242 studies,^193^ an evaluation was conducted on three commercially available, non-additive manufacturing fabricated TMJ implants, including the patient-tailored TMJ Concepts implant, the Nexus CMF system, and the stock and customized Biomet implants. The analysis revealed no significant differences in outcomes related to pain and dietary restrictions among the implants produced by these manufacturers. Nonetheless, these TMJ systems vary significantly in material composition, implant design, manufacturing methods, preclinical testing, regulatory approval status, and clinical outcome reporting.^194^ It is crucial, therefore, to ensure that all current and future TMJ protheses undergo rigorous scientific validation to guarantee their safety and effectiveness.

#### Application in skeletally immature patients

The prevailing consensus in reconstructive surgery has traditionally favored autogenous materials for pediatric cases and alloplastic materials for adults. By ensuring that the facial skeleton has largely completed its growth, this age-specific approach minimizes the risk of ongoing skeletal changes compromising the effectiveness and longevity of the reconstruction. However, given the potential complications associated with autogenous grafts in children—such as interference with growth—and the documented success of alloplastic TMJ prostheses, it is becoming increasingly reasonable to explore alloplastic reconstruction in select pediatric populations.^122,195–197^ To mitigate concerns related to growth interference and other complications, several strategies could be conducted. These include comprehensive preoperative planning and customization, multidisciplinary collaboration, precise prosthesis design and positioning, meticulous surgical methods, and the application of interposition spacer materials. Moreover, it is also necessary and important for postoperative monitoring with regular follow-up. Ensuring accurate placement of the prosthesis is essential for maintaining joint biomechanics and balancing the tension between the reconstructed joint and the surrounding structures, such as the maxilla.^198^

Several studies have suggested that the use of alloplastic materials in skeletally immature patients does not adversely affect mandibular growth or the patient’s ability to achieve improved maximum incisal opening following bilateral or unilateral TMJR implantation.^195,197,199,200^ Among these studies, Douglas utilized alloplastic total TMJ reconstruction for two 4-year-old children with ankylosis and followed them for more than 8 years.^197^ Similarly, Keyser conducted a pilot survey on the application of alloplastic TMJR for 14 growing patients with follow-ups extending up to 10 years.^195^ The results of these studies showed that none of the alloplastic joints required replacement or explanation. In addition, following alloplastic joint replacement, mandibular growth continued and was not entirely halted. There was a consistent and substantial improvement in MIO over the long term, accompanied by improvements in overall mandibular functions such as speech and mastication. A recent systematic review summarized the current application of alloplastic TMJR in skeletally immature patients. It included a total of 73 skeletally immature patients from 7 countries who underwent total alloplastic TMJR.^198^ The review indicated that all patients had undergone multiple surgeries before the application of alloplastic total TMJ reconstruction. The included studies demonstrated significant enhancement in MIO and improvements in mandibular function during follow-up.

These findings suggest that alloplastic TMJ reconstruction can be a viable and effective option for pediatric patients, offering long-term benefits in joint function and overall quality of life. Despite this, half of the patients had less than three years of follow-up, highlighting the necessity for further long-term clinical research into the benefits of alloplastic TMJ prostheses in pediatric populations. In summary, the use of alloplastic TMJR is a controversial treatment option for skeletal immature patients and might be recommended only in the most difficult cases. This method may be reserved for treating refractory ankylosis or following multiple unsuccessful attempts to repair the ankylosed joint. It is important to note that many children with TMJ ankylosis already lack the mandibular growth potential seen in children without the condition. Ideally, the placement of alloplastic TMJR should be delayed until late adolescence or adulthood to ensure that the majority of the patient’s skeletal growth is complete.

### Postoperative complications of alloplastic TMJR

Alloplastic TMJR, while beneficial, is not devoid of risks. Short-term complications may include facial nerve weakness, infection, metal hypersensitivity, and postoperative malocclusion. Long-term challenges encompass implant instability, loosening of screws, relapse of TMJ ankylosis, and unresolved functional deficits, potentially necessitating device revision or replacement.^6,154,181,201,202^

#### Facial nerve injury

Facial nerve weakness is the most common complication associated with TMJR, with manifestations ranging from paresis and paralysis (7.8%) to sensory alterations (1.8%).^189^ The proximity of the surgical site for TMJR installation to vital structures and the prolonged retraction of tissues, which may stretch and temporarily impact nerve function, likely contribute to these outcomes.^147,153,177,178,185,190,203–209^ In most studies, transient weakness of the temporal, buccal, and marginal mandibular branches of the facial nerve is observed immediately postoperatively and typically resolves within six months.^147,210^ A Although a minority of patients experience persistent paralysis of the temporal branch necessitating a unilateral brow lift, the risk of permanent facial nerve damage remains very low.^147,207^ Further investigations have identified relatively predictable factors that increase the risk of temporary facial nerve injury, including revision TMJ replacement, bilateral surgery, and multiple open TMJ procedures. In contrast, the risk factors for permanent injury are less predictable but are likely similar.^211^ Larger clinical studies are needed to elucidate specific risk factors definitively.

We advocate for the routine identification of facial nerve branches in the operative field. This practice not only guides the dissection process but also ensures that the nerve’s anatomical integrity is confirmed by the end of the surgery, offering reassurance to both patient and physician in cases of postoperative facial nerve dysfunction. Careful dissection along fascial planes is essential to prevent nerve injury. Extreme caution must be exercised during nerve dissection, particularly in revision surgeries where scar tissue may obscure visualization and increase the risk of nerve damage. The preauricular approach has been reported to provide better access with a reduced risk of facial nerve injury.^147^ Notably, the most frequent surgical procedures associated with facial nerve injury are oral and maxillofacial surgeries, especially TMJ replacement operations, which account for 40% of such injuries.^212^ In addition, the application of low-intensity laser therapy, particularly when augmented with vitamin complex medication, has demonstrated efficacy in mitigating these effects.^189^

#### Infection

The incidence of surgical site infection (SSI) following TMJR is relatively low (0.7%).^189^ However, when SSI do occur, the clinical and economic consequences can be significant. These infections may arise through hematogenous spread or localized introduction during surgery,^145,154,177,178,204,213–217^ and can manifest over a mean period of 6 months postoperatively, with a range of 2 weeks to 12 years. Several host comorbidities have been reported and should be assessed and managed preoperatively to reduce the risk of SSI. These factors include metabolic diseases (e.g., diabetes), high inflammatory arthritis, anxiety and depression, use of immunosuppressive medications, malnutrition, cardiac and pulmonary diseases, anemia, and HIV/AIDS. In addition, nicotine use (with cessation recommended 4 to 6 weeks before surgery), alcohol and drug abuse are also significant factors.^218^

It is noteworthy that a recent retrospective study spanning over 20 years found that the most commonly cultured organisms in prosthetic joint infections (PJI) of the TMJ were *Staphylococcus aureus* (53%), with *Propionibacterium acnes* colonization noted in 33% of cases.^219^ Consequently, several key strategies can be applied to prevent SSI and PJI. These include reducing patients’ bacterial burden through antimicrobial photo-disinfection therapy combined with chlorhexidine gluconate body wipes,^220^ administering prophylactic antibiotics (1st- or 2nd-generation cephalosporins were recommended) one hour prior to surgery,^221^ developing innovative coatings to confer potential antibacterial activity on the TMJ implant surfaces,^222,223^ and establishing an optimal surgical environment by implementing routine preoperative bathing, avoiding preoperative hair removal, and soaking prosthetic components in antibiotic solutions.^221,224^

Prevention remains the most effective strategy; however, making a timely and accurate diagnosis of PJI is crucial for successful and targeted management. It could be challenging to distinguish a PJI from an adverse local tissue reaction to particulate wear without the presence of purulence.^225^ Culture-negative PJI infections occur in 27% to 55% of cases, often due to biofilms that are not easily identified with conventional culture methods. To enhance culture yield, it is recommended to withhold antibiotics before taking culture samples, culture synovial fluid in blood culture bottles, and extend the culture duration.^226^ The latter is particularly relevant when dealing with *Propionibacterium acnes* PJI.^227^ Recently, emerging techniques such as the leukocyte esterase test,^228^ diagnostic tests for interleukin 6,^229^ Alpha-defensin^230^ and Serum D-dimer,^231^ and next-generation sequencing^232^ have shown high sensitivity and specificity and are becoming feasible in clinical settings.

A 7–10 day course of oral antibiotic prophylaxis is recommended as a postoperative intervention following TMJR, due to the surgical wounds’ proximity to potential contamination sources such as the ear, parotid gland, and oral cavity.^218^ Effective management strategies include the early administration of broad-spectrum antibiotics and surgical intervention for drainage, ideally within five days of symptom onset. In cases where infection persists, reconstruction with a new prosthesis, accompanied by an autogenous fat graft around the implant site, is recommended after a period of 8–10 weeks, if deemed necessary.^216^ In addition, because the condylar component ramus fixation screws are positioned in the pterygomandibular space and may become contaminated during the administration of inferior alveolar nerve anesthesia, prophylactic antibiotics are recommended for patients undergoing inferior alveolar nerve blocks.^224^

#### Metal hypersensitivity

Metal hypersensitivity can develop at any age and has a significantly higher incidence in females.^233^ Chronic exposure to low concentrations of metal ions or particles, or acute exposure to high concentrations from dissolution, corrosion, or wear, can induce metal hypersensitivity.^234^ Metal wear debris acts as haptens, triggering allergic sensitization through processing by antigen-presenting cells. Notably, while metal-on-metal TMJ prostheses exhibit reduced wear, they are associated with a higher incidence of metal hypersensitivity compared to metal-on-UHMWPE systems. Current estimates indicate that approximately 10% to 15% of the population may exhibit an allergy to one or more metals commonly used in implantology.^235,236^ Symptoms of hypersensitivity reactions can range from local (such as skin dermatitis and erythema) to systemic effects (including neurological or gastrointestinal issues).^139^ Common cutaneous reactions associated with metallic implants include vasculitis, dermatitis, eczema, and occasionally urticaria. In certain cases, these local reactions can cause the implant to loosen, ultimately leading to failure.^237^

Metallic biomaterials, including Co–Cr and Ti alloys, are generally biocompatible due to the formation of protective oxides like Cr_2_O_3_ (in Co–Cr alloys) or TiO_2_ (in Ti alloys). Patients with documented hypersensitivity to Co–Cr–Mo alloy who require TMJ replacement have been reported to experience significant improvements in jaw function, diet, TMJ pain, jaw opening, headaches, disability, and quality of life when the mandibular components are made from all-Ti alloy.^238^ However, trace elements such as Nickel (Ni), Aluminum (Al), Vanadium (V), and Titanium (Ti) may also elicit allergic reactions.^236^ To mitigate allergic reactions and reduce the potential risk of initial prosthesis rejection, pre-implantation screening via skin patch tests or lymphocyte transformation tests is recommended,^139,236,239^ particularly for patients with a history of intolerance to jewelry, belt buckles, watches, or a prior metal implant. The lymphocyte transformation test measures lymphocyte proliferation in the presence and absence of a metal ion stimulus when cultured with peripheral blood lymphocytes. Researchers have used lymphocyte transformation tests to assess patients with symptomatic orthopedic implants who had negative skin patch tests, thereby identifying patients who might benefit from implant removal.^240,241^ Despite the availability of laboratory tests to evaluate patients for potential metal allergy, no consensus was obtained on the optimal timing or specific clinical situations for evaluating patients for metal allergy or hypersensitivity.

In cases of positive hypersensitivity, the use of an allergen-free prosthesis is advised. For patients displaying hypersensitivity symptoms postoperatively, initial conservative management is recommended. To alleviate hypersensitivity symptoms, the use of antihistamines and short-term courses of topical or systemic corticosteroids is usually recommended.^242^ If this approach is unsuccessful, a lymphocyte transformation test should be conducted. A positive test result mandates prosthesis replacement, while a negative result calls for ongoing observation to determine whether prosthesis retention or removal is appropriate.^243^

#### Heterotopic ossification

Heterotopic ossification (HO), as detailed in orthopedic literature, denotes the aberrant formation of ectopic bone within soft tissues or joints.^244^ HO is classified into two primary types: acquired and hereditary. Acquired HO, the more common variant, is associated with diverse etiological factors including trauma, fractures, surgical interventions, soft tissue damage, burns, infections, arthritis, and neurogenic injuries.^245^ Particularly in the context of alloplastic TMRJ for managing TMJ ankylosis, recurrent acquired HO (1%) and re-ankylosis pose significant challenges,^145,148,178,185,187,217^ potentially leading to pain and restricted mandibular function.^246,247^

Recent advances have highlighted the efficacy of abdominal fat grafting in obliterating dead space and preserving adequate space for TMJR, alongside perioperative radiation, in mitigating the risk of heterotopic bone formation.^218,246,248,249^ In addition, the critical role of outpatient follow-up with daily physical therapy for at least six months cannot be overstated, as it is pivotal in promoting mandibular mobility.^247^ In instances where HO is diagnosed, surgical exploration and debridement of the heterotopic bone are recommended as effective interventions.^246,247,250^

#### Prosthesis dislocation

Dislocation of the prosthesis is a noted complication in TMJR, as observed in five studies.^203,206,209,251^ Particularly, the TMJ prothesis is susceptible to dislocation, primarily within the initial six weeks postoperatively.^251^ Contributing factors to prosthesis dislocation include insufficient muscular stability, sectioning of the pterygomasseteric sling, inadequate adaptation of prosthetic components, and removal of the coronoid process. Anterior dislocation occurs due to incorrect positioning of the condyle/fossa component and can result from releasing the masticatory muscles and simultaneous coronoidectomy.^209,252^

Misalignment of the stock condyle in the center of the fossa can lead to posterior displacement, causing impingement on the tympanic plate or auditory canal, resulting in pain, mandibular dysfunction, and potential infection due to pressure-related perforation.^253^ Conversely, the custom-made prostheses often incorporate a posterior stop on the fossa component to prevent posterior displacement of the condyle component, alleviating this concern. However, this preventive feature may be absent in some stock prostheses, increasing the risk of the condyle component displacing posteriorly if not precisely centered within the fossa.^253^

Post-surgical dislocation necessitates prompt intervention, typically involving physiotherapy and the application of intermaxillary elastics to stabilize the prosthesis for at least one week. Early postoperative dislocations can often be resolved by repositioning the ramus component followed by intermaxillary elastics.^206,209,252^ However, in certain cases, repositioning under general anesthesia or light sedation may be required to address the dislocation effectively.^209^

## The future of alloplastic TMJR

### Emerging materials in TMJ reconstruction

#### Co–Cr alloys

Co–Cr alloys have historically been favored in the manufacture of load-bearing total joint implants, including TMJR devices. This preference is attributed to their combination of high strength, superior wear and fatigue resistance, and notable biocompatibility, the latter of which is largely due to a passivating chromium oxide layer.^143,253^ Subsequent developments led to the introduction of a wrought ASTM F1537 Co–Cr–Mo alloy, with compositions ranging from 58.9 to 69.5 wt% Co, 27.0 to 30.0 wt% Cr, 5.0 to 7.0 wt% Mo, and up to 1 wt% Ni. This alloy, boasting enhanced mechanical properties and wear resistance, received FDA approval for use in TMJR devices.^141,187^ However, the presence of residual Ni has raised concerns regarding material hypersensitivity, and the animal studies conducted by McGregor et al. have suggested carcinogenic potential associated with metallic Co and Co alloys.^239,254^

In response to these concerns, research efforts have pivoted towards developing Co- and Ni-free alloys that maintain comparable biological and bioengineering characteristics. Initial studies identified Fe24Cr2MoN, a high nitrogen nickel-free austenitic stainless steel, as a potential alternative.^255,256^ Despite its promising attributes, this material demonstrated susceptibility to wear, pitting, and fretting corrosion in simulated body fluid environments, leading to concerns over material integrity and the release of corrosion products.^257^ A breakthrough came with Radice et al.’s investigation into a novel nickel-free high nitrogen stainless steel variant, Fe18Cr14Mn3.5MoN0.9. This new composition exhibited significantly higher corrosion resistance in comparison to its predecessors under analogous bovine serum testing conditions,^258^ marking a significant advance in the search for safer, more durable materials for TMJR devices.

#### Titanium alloys

Co–Cr–Mo alloys have historically been the cornerstone in the development of load-bearing joint implants due to their robust mechanical properties and biocompatibility. However, escalating concerns regarding the stress shielding effects and potential toxicity associated with Co–Cr alloys have catalyzed the shift towards Ti alloys in TMJR applications.^139,238^ The superior passivating ability of the titanium oxide layer significantly reduces metal ion release compared to its Co–Cr and stainless-steel counterparts, thereby minimizing adverse tissue reactions.^170,253^ This attribute has made Ti alloys particularly beneficial for patients with known hypersensitivity to Co–Cr–Mo, with reported improvements in TMJ pain, functionality, and overall quality of life following treatment with Ti-based TMJR devices. Among the Ti materials, commercially pure titanium (Cp Ti, 98.8 wt%–99.6 wt% Ti) and Ti–6Al–4V (89.0 wt%–91.0 wt% Ti, 5.5 wt%–6.5 wt% Al, and 3.5 wt%–4.5 wt% V) are predominant, both receiving FDA approval for use in TMJR due to their optimal blend of biocompatibility and mechanical strength.^259^ Ti–6Al–4V, an alloy containing both α- and β-phases, is known for its enhanced tensile and fatigue strength, attributable to thermomechanical processing.^170^ Conversely, Cp Ti, composed solely of the α-phase, exhibits lower mechanical strength but boasts superior corrosion resistance due to the lack of alloying elements in its protective oxide layer, rendering it highly biocompatible.^235^

While Ti–6Al–4V has been a predominant alloy in TMJR owing to its excellent mechanical properties and biocompatibility, concerns regarding the long-term release of aluminum and vanadium—and their potential to induce hypersensitivity—have prompted research into alternative titanium alloys.^260^ This has led to the development of novel beta-Ti alloys,^261^ such as Ti-Zr-Mo-Fe and Ti-Nb-Zr-Ta, which incorporate nontoxic elements like tin (Sn), zirconium (Zr), tantalum (Ta), molybdenum (Mo), and niobium (Nb) to achieve similar or superior mechanical and clinical properties.^170,259,262^ These innovative beta-Ti alloys are heralded for their lower elastic modulus, which theoretically reduces stress shielding at the implant-bone interface—a critical factor in the longevity and success of an implant. The inclusion of elements like Nb, Zr, and Ta not only contributes to this reduced modulus but also facilitates the formation of more stable and protective oxide layers (e.g., Nb_2_O_5_, ZrO_2_, or Ta_2_O_5_), enhancing the corrosion resistance and biocompatibility of the implants.^262^

Despite the promising characteristics of beta-Ti alloys in TMJR applications, their comparatively lower fatigue strength relative to Ti–6Al–4V has raised concerns regarding their suitability for articulating joint surfaces.^139,262^ This limitation underscores the need for alloy modification to enhance mechanical robustness while maintaining or improving biocompatibility. Recent advancements have demonstrated that targeted modifications, such as laser gas alloying with nitrogen and the incorporation of iron (Fe) and silicon (Si) into the beta-Ti alloy matrix (e.g., Ti-35Nb-7Zr-6Ta-2Fe-0.5Si), can significantly bolster both mechanical and biological properties of these materials.^262,263^ Furthermore, the interface between TMJR devices and UHMWPE components has been a focal point for reducing wear and enhancing corrosion resistance. Studies have shown that diamond-like carbon (DLC)-coated stainless steel and titanium, when paired with UHMWPE, exhibit markedly reduced wear and superior corrosion resistance compared to their uncoated counterparts.^264–266^

#### Polyethylene

Despite recent advancements in the development of Ti alloys, their mismatch in elastic modulus with bone tissue continues to pose significant challenges in orthopedic applications. This limitation has spurred interest in non-metallic fiber-reinforced composites as potential alternatives for load-bearing implants, offering a closer match to bone’s mechanical properties.^267^ Since its initial application in orthopedic surgery in 1962, UHMWPE has emerged as the predominant bearing surface material in total joint replacement devices.^268^ Characterized by its linear, unbranched structure, high molecular weight, and substantial crystallinity, UHMWPE offers enhanced wear resistance and reduced friction coefficients when comparing to other polymers such as high-density polyethylene, polymethyl methacrylate, and polytetrafluoroethylene.^269^ Over five decades, advancements have culminated in the development of high-grade cross-linked UHMWPEs, marking a significant improvement in wear resistance and wear rates over earlier formulations.^270^ Recent studies report success rates ranging from 84% to 91% for TMJR employing UHMWPE fossa, highlighting its efficacy and durability in clinical applications.^141,149^

Initial apprehensions regarding the use of UHMWPE in tibial liners centered on potential embrittlement and an increased fracture risk. However, the functional loads exerted on knee and hip prostheses significantly surpass those on the TMJ, substantially mitigating concerns about polyethylene wear and fracture risks in TMJR.^271,272^ Notably, Wolford’s studies revealed that cases using metal-on-metal TMJ devices showed markedly higher systemic levels of Cr and Co, alongside a greater prevalence of metal hypersensitivity, compared to those with metal-on-UHMWPE prostheses.^234^ Despite the increased wear observed with metal-on-UHMWPE implants relative to metal-on-metal prostheses employing Co–Cr–Mo alloys, this issue can be effectively managed by augmenting the thickness of the articulating surface, as demonstrated by the Biomet TMJ prosthesis, which features a minimum UHMWPE fossa thickness of 4 mm.^149^ Nonetheless, long-term follow-ups identified potential issues such as creep^269^ and shelf aging^273^ with UHMWPE in TMJR, potentially leading to increased micromotion and eventual device failure. These challenges have been partially addressed by integrating vitamin E into UHMWPE or blending α-tocopherol, enhancing the mechanical strength and reducing deterioration of the material, thereby presenting a promising avenue for improving the longevity and performance of TMJR devices.^187,273^

Investigations into ceramics such as Al_2_O_3_ and ZrO_2_,^274^ polyetheretherketone (PEEK)^275^ and DLC^259^ have expanded the repertoire of materials considered for bearing surfaces in hip and knee total joint replacement systems. Among these, ceramic materials, notable for their superior tribological performance, offer significant advantages over metals and polymers. Specifically, zirconia-toughened alumina (ZTA) composites, which combine Al_2_O_3_ in the primary phase (70–95%) with ZrO_2_ in the subsequent phase (5–30%), have been highlighted for their exceptional aging and wear resistance. The integration of ZrO_2_ not only preserves the inherent strengths of the Al_2_O_3_ matrix but also enhances the composite’s strength and fracture toughness.^276^ Recent advancements in ZTA materials, featuring a nano-sized microstructure, have demonstrated limited wear damage and outstanding crack resistance in hip simulators, suggesting their potential suitability as articulating bearing surfaces in TMJR systems.^277^ Conversely, studies indicate that PEEK and carbon fiber-reinforced PEEK exhibit significantly higher wear rates than UHMWPE, casting doubts on their viability as bearing surfaces for TMJR systems.^278,279^ Despite these advancements, the scarcity of data within the craniomaxillofacial surgery domain underscores a critical need for further research and development. This endeavor is crucial to ensure the safety and efficacy of new materials in TMJR applications, thus calling for a concerted effort to fill this gap in our current understanding.

### Additive manufacturing techniques used in TMJ reconstruction

**Fig. 8:**
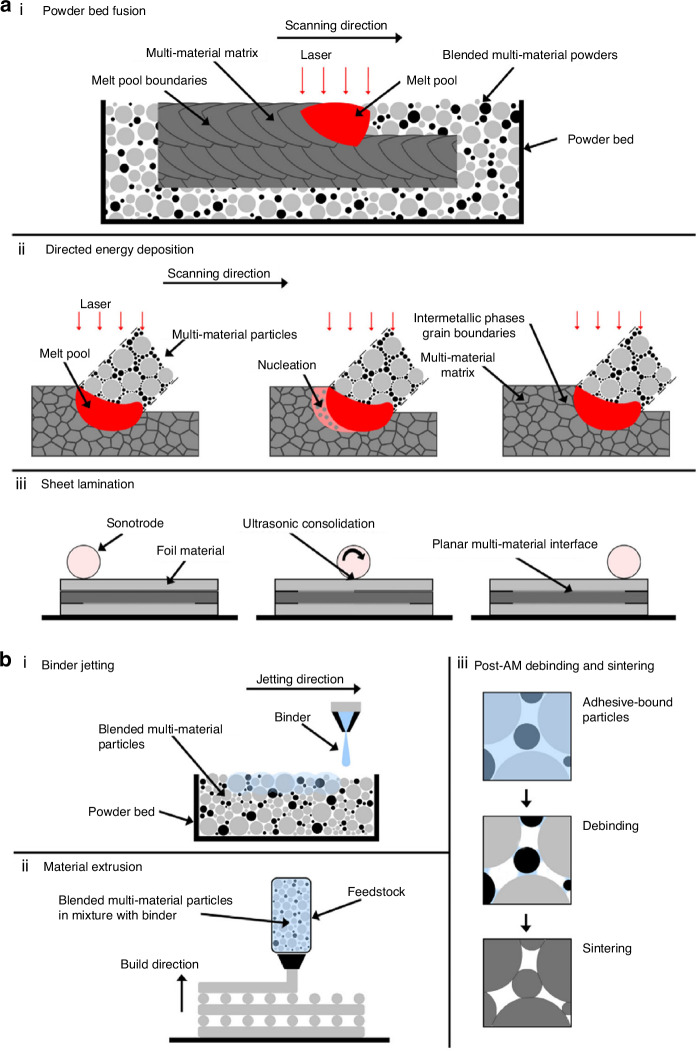
Additive manufacturing technologies used for Ti-based biomaterials for bone substitution. **a** Laser and ultrasonic multi-material AM for metals according to the process classifications of ASTM F2792-12a. **b** Adhesive multi-material AM for metals according to the process classifications of ASTM F2792-12a.^281^ Copyright © 2020 The Authors

**Fig. 9:**
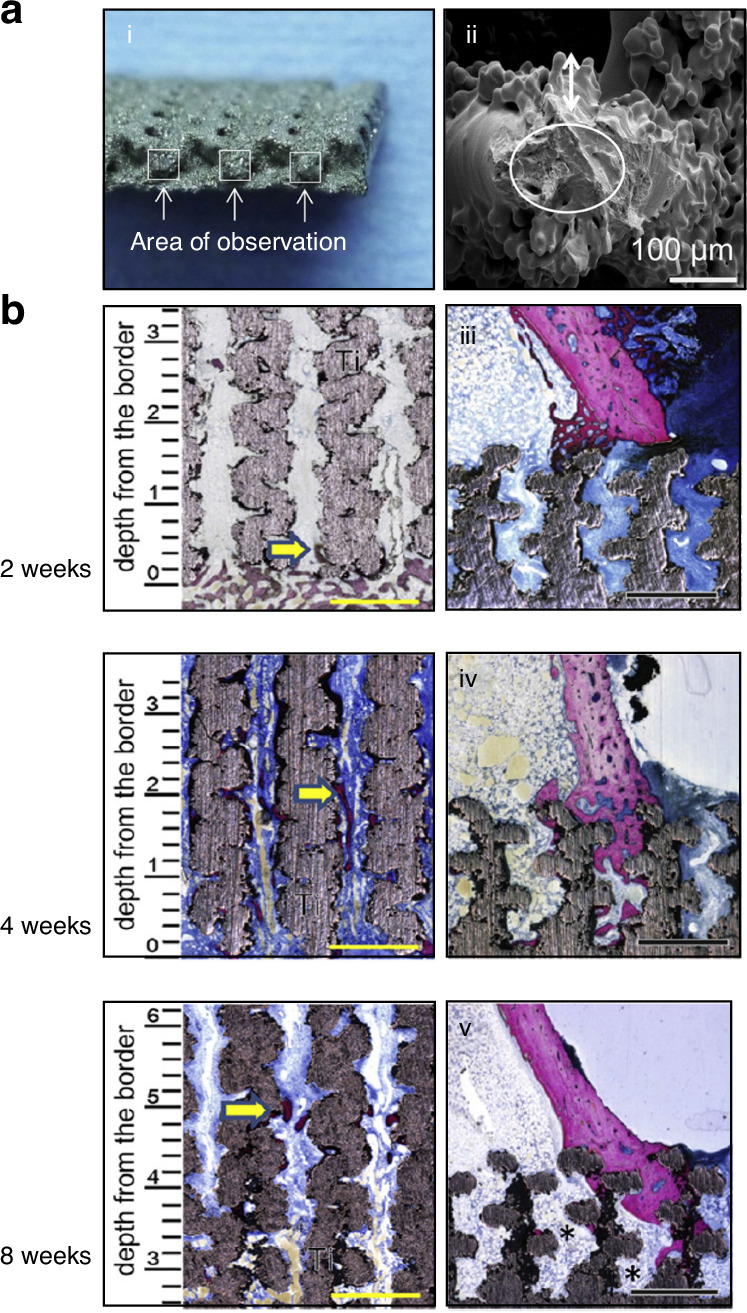
Additive manufacturing technologies used for porous-surfaced titanium plates. **a** Scanning electron microscopy images of the cross section of the inner strut of porous titanium-surfaced plate. **b** Non-decalcified histologic sections of porous-surfaced titanium plates implanted into rabbit tibia. Stain: Stevenel’s blue and Van Gieson’s picrofuchsin. Purple indicates bone; silver indicates the titanium implant. *: marrow-like tissue spreading into porous area. Scale bars: 1 mm.^283^ Copyright © 2015 Elsevier B.V

Recent advancements in additive manufacturing (AM), also known as 3D printing, have notably enhanced the production of TMJR devices. These advancements offer several benefits, including improved metal porosity and expedited production timelines. AM refers to creating three-dimensional objects by sequentially adding material in layers,^280^ which primarily employs metal powder bed fusion (PBF) techniques for the fabrication of TMJR, including selective laser sintering, selective laser melting, direct metal laser sintering (DMLS), and electron beam melting. These techniques have been shown to provide superior mechanical properties and biocompatibility for TMJR^281^ (Fig. 8a, b). Specifically, PBF processes involve melting or sintering powder layers using a focused energy source, such as an electron or laser beam, facilitating the creation of complex structures characterized by high precision and optimal porosity. This approach offers unparalleled design flexibility, enabling the production of complex, patient-specific structures that precisely conform to an individual’s mandible, free from the limitations of conventional tooling.^282^ Moreover, AM enables the fabrication of porous TMJ implants with meticulously controlled pore sizes, porosity levels, and interconnectivity (Fig. 9a). This design feature promotes bone ingrowth and enhances drug delivery while ensuring optimal permeability and diffusivity^283^ (Fig. 9b). The technology also allows for the integration of components with varying mechanical properties within a single implant structure. The mechanical characteristics can be precisely modified through topological optimization of the porous structure to closely resemble the replaced bone, thus minimizing the risk of stress shielding.^284^

**Fig. 10:**
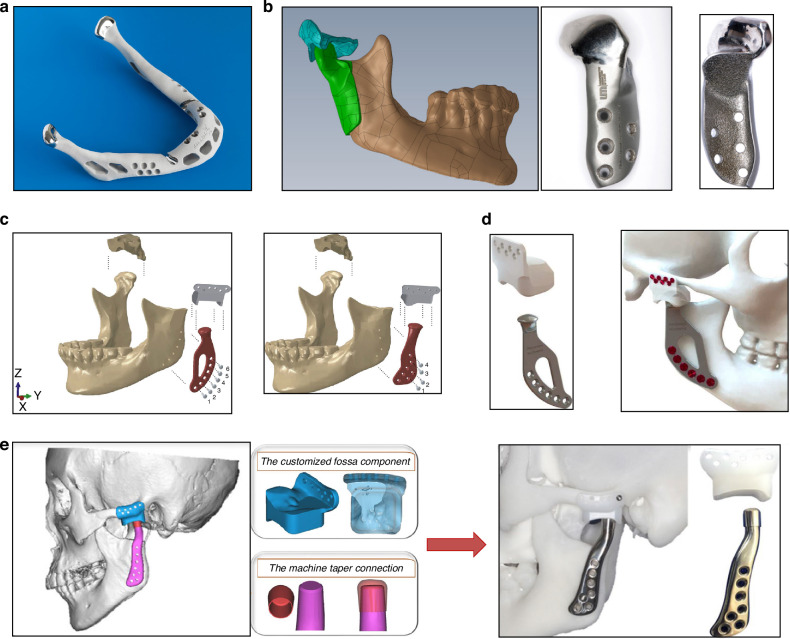
Current applied additive manufacturing TMJ prothesis. **a** The first patient-specific entire lower jaw AM replacement.^290^ Copyright © 2012 Elsevier Ltd. **b** Virtual model of a customized AM implant made using a CAD system (Left). Customized TMJ implants made of a titanium alloy and fabricated by using AM (DMLS), showing holes for fixing screws and for muscle attachments (Right).^289^ Copyright © 2017 European Association for Cranio-Maxillo-Facial Surgery. **c** Melbourne prosthetic TMJ and Biomet Microfixation prosthetic TMJ developed by the researchers of the University of Melbourne and used in the study of Ackland et al.^285^ Copyright © 2017 Elsevier Ltd. **d** The TMJ prosthetic total joint replacement system developed by OMX Solutions and used in the study of Dimitroulis et al. The 3 d printing TMJ prosthetic total joint replacement system is composed of an Ultra-high molecular weight Polyethylene Fossa and a Titanium Alloy condylar ramus unit (left) that are secured to the bone with titanium alloy screws (right).^287^ Copyright © 2018 European Association for Cranio-Maxillo-Facial Surgery. **e** The processing of the new TMJ prosthesis used in the study of Zheng et al., including the pre-processing for the craniomaxillofacial model, the design for the prosthesis, and the manufacture for the prosthesis.^291^ Copyright © The Author(s) 2019

AM has emerged as a particularly advantageous method for crafting patient-specific medical devices, such as TMJ implants. This preference stems from AM’s flexibility in producing single or small batches of items, making it ideally suited for custom-designed medical implants tailored to individual patients’ anatomical requirements. Such precision ensures a near-perfect fit, significantly enhancing the effectiveness of TMJ reconstruction.^285–289^ The transformative potential of AM in the medical field was starkly illustrated in 2012 with the first clinical application of an AM-produced TMJ implant, which involved the complete replacement of a patient’s lower jawbone^290^ (Fig. 10a). This landmark procedure underscored AM’s capability to produce highly complex, anatomically precise implants. Currently, ~27% of TMJR devices produced globally incorporate components manufactured via additive processes, reflecting the growing recognition of AM’s value in this domain.^268^ The primary benefits of AM for custom TMJ prostheses, as corroborated by multiple studies, include the production of implants that provide a secure and comfortable fit (Fig. 10b–e). This is achieved through AM’s ability to fabricate devices that accurately conform to the unique contours of a patient’s mandible, offering an alternative to one-size-fits-all solutions. In addition, AM’s capacity to rapidly transform intricate designs into physical products at a reasonable cost has been highlighted as a significant advantage.^291,292^ A noteworthy study that compared AM with traditional manufacturing techniques for TMJ implants found no statistical difference in functional outcomes post-surgery, affirming the safety and efficacy of AM-fabricated devices.^289^ An in vitro study compared 3D-printed titanium (3D-Ti) plates with standard Synthes-Ti plates. The results demonstrated that 3D-Ti plates offer similar biocompatibility and stability for rigid internal fixation, while also exhibiting lower surface roughness, superior mechanical strength, and a higher bone–plate contact rate.^293^ Moreover, A recent systematic review and meta-analysis compared the mechanical and biological properties of resin materials, including PEEK, used in AM techniques for fabricating oral appliances, with those of conventionally manufactured materials. The results demonstrated that 3D-printed prothesis exhibited satisfactory mechanical performance compared to conventional approach.^294^ This finding reinforces the position of AM as a viable and promising approach to produce TMJR devices, potentially revolutionizing patient-specific treatment strategies.

Despite the considerable advantages offered by AM in producing patient-specific TMJR devices, several technical challenges inherent to the process warrant attention.^295^ These challenges include deformation, warping, and cracking of the final product, which may be primarily attributed to the differential melting and cooling mechanisms characteristic of AM. These issues arise from significant heat transfer, rapid cooling rates, and potentially suboptimal manufacturing parameters prevalent in 3D printing processes. Consequently, 3D-printed alloys are often reported to possess inferior corrosion resistance when compared to their wrought counterparts.^296^ In addition to these technical hurdles, AM faces other limitations that can impact its broader adoption for medical applications. These include constraints on part sizes, subpar surface finishes, the high cost of certain AM machinery, the necessity for specialized software—which may incur additional expenses—and the limited availability of suitable starting materials.^280^ While these challenges pertain mostly to the manufacturing process itself, it’s imperative to acknowledge that the clinical efficacy and benefits of AM-fabricated TMJ prostheses remain underexplored in the current literatures. Currently, the field of metal AM, particularly for TMJR applications, is still evolving. A deeper understanding of the interplay between processing conditions, microstructure, and material properties is crucial for advancing this technology.^268^ The current state of knowledge underscores the necessity for further clinical research to substantiate the superiority of AM over conventional manufacturing techniques for TMJR devices. More comprehensive clinical outcome data are essential to conclusively demonstrate the efficacy and reliability of AM in this context.

## Conclusion

Reconstruction of the TMJ represents a niche yet profoundly impactful challenge within the realm of head and neck surgery, significantly affecting patients’ functionality and quality of life. Due to its infrequent occurrence and the complex etiology encompassing trauma, degeneration, and congenital defects, TMJ reconstruction lacks a unified approach, resulting in considerable variability in clinical practice. Current methods for TMJR range from autologous grafting to alloplastic joint replacement, each offering distinct advantages and limitations based on the specific patient situations. This variability emphasizes the urgent necessity for establishing a consensus on the most effective reconstruction strategies to meet the distinct requirements of individual cases.

In the author’s opinion, alloplastic joint replacement, particularly custom alloplastic TMJR, has become the preferred method and is increasingly regarded as the gold standard for reconstructing end-stage TMJ disorders, especially in skeletally mature patients. Current prostheses now have up to 20 years of follow-up data, demonstrating favorable short-, medium-, and long-term outcomes. However, it remains uncertain whether these outcomes will be sustained beyond 20 years. Advances in the design and materials of TMJ prostheses, such as the use of biocompatible materials, have further minimized the risk of rejection and complications, enhancing both the longevity and functionality of the joint replacement. Although the initial work-up for these prostheses, including 3D CT scans and models, is more extensive, the benefits—such as reduced operative time, shorter hospital stays, and fewer secondary donor site complications—far outweigh the initial cost of the prosthesis. For TMJ reconstruction in pediatric patients, however, CCG remains the preferred option due to its growth potential. Successful free grafting depends on a well-vascularized bed, and scarred tissue, with reduced vascularity, may compromise graft viability. DO and vascularized free flaps are typically considered in revision surgeries when the soft tissues fail to provide an adequate vascular bed for non-vascularized tissue transfers.

Currently, UHMWPE remains the ‘gold standard’ bearing surface for orthopedic joint replacement devices, particularly in hip and knee replacements. For major components of orthopedic and dental replacement devices, Ti6AL4V alloy is the preferred metal due to its biocompatibility and excellent bio-integration. However, despite its high strength and corrosion resistance, studies have detected titanium wear particles and ions in local peri-implant tissues as well as in distant organs. To address some of the drawbacks of titanium, advanced technologies like CAD/CAM, 3D printing, and VSP have revolutionized TMJ reconstruction. These technologies enable the production of custom-fitted prostheses that precisely match individual patient anatomy, leading to improved surgical accuracy, shorter recovery times, and higher patient satisfaction. Future developments in TMJR devices must focus on ensuring that compounds or coatings designed to combat biofilm formation are properly applied to surfaces to prevent wear over time, while also ensuring the effective delivery of anti-biofilm agents. As 3D printing continues to evolve, the production of TMJR systems may become more efficient and cost-effective.

Despite notable advancements in TMJR through alloplastic prostheses, challenges persist that are worth attention. Among these considerations, the necessity for comprehensive long-term studies is particularly pronounced, especially aimed at clarifying outcomes for pediatric patients. The intrinsic growth dynamics of pediatric patients bring complex variables into both the integration and long-term performance of alloplastic prostheses. Moreover, there is a pressing need for innovation in surgical methodologies and AM of biomaterials to minimize complications and expedite recovery. Ongoing investigations are crucial to address these challenges, with a continued focus on improving the quality of life for patients with end-stage TMJ disorders. At this stage, aside from the initial costs, there appears to be little justification for considering alternative forms of reconstruction in adults. However, the development of custom-made cartilage grafts using stem cells may represent the future of joint reconstruction across various types.

In summary, the field of TMJ reconstruction has witnessed remarkable progress, moving towards more reliable, less invasive, and more patient-specific treatments. The future of TMJ reconstruction lies in the refinement of these innovative technologies and methods, along with a deeper understanding of the TMJ’s biological and mechanical behaviors and its pathological conditions. Current reconstructive techniques favor autogenous replacements in children and alloplastic replacements in adults. However, the trend is gradually shifting towards the use of alloplastic TMJR in older children. The integration of advanced materials, personalized prosthetics, and cutting-edge manufacturing techniques will continue to drive the field forward, addressing both current challenges and future needs in TMJ reconstruction.

## Data Availability

The data that support the findings of this study are available from the corresponding author upon reasonable request.
